# Actual status of patient information sharing among healthcare delivery facilities: a survey by the third subcommittee, committee on academic research, the Japanese society of hospital pharmacists

**DOI:** 10.1186/s40780-022-00260-z

**Published:** 2022-11-04

**Authors:** Daisuke Kikuchi, Masami Tsuchiya, Shiro Hatakeyama, Yuichi Tasaka, Takeshi Uchikura, Ryohkan Funakoshi, Taku Obara

**Affiliations:** 1 Department of Pharmacy, Tohoku Medical and Pharmaceutical University Hospital, 1-12-1 Fukumuro, Miyagino-ku, 983-8512 Sendai, Miyagi Japan; 2 Committee on Academic Research, Third Subcommittee, the Japanese Society of Hospital Pharmacists, 2-12-15, Shibuya, Shibuya-ku, 150-0002 Tokyo, Japan; 3 Department of Pharmacy, Miyagi Cancer Center, 47-1 Nodayama, 981-1293 Medeshimashiote, Natori, Miyagi Japan; 4 Division of Pharmacy, Yamagata University Hospital, 2-2-2 Iida-nishi, 990-9585 Yamagata-shi, Yamagata, Japan; 5 Laboratory of Clinical Pharmacy, School of Pharmacy, Shujitsu University, 1-6-1 Nishigawara, Naka-ku, 703-8516 Okayama, Okayama Japan; 6 Department of Hospital Pharmaceutics, School of Pharmacy, Showa University, 1-5-8 Hatanodai, Shinagawa-ku, 142-8666 Tokyo, Japan; 7 Department of Pharmacy, Kameda General Hospital, 929 Higashi-cho, 296-8602 Kamogawa-City, Chiba Japan; 8 Department of Pharmaceutical Sciences, Tohoku University Hospital, 1-1, Seriyo-machi, Aoba- ku, 980-8575 Sendai, Miyagi Japan; 9 Division of Preventive Medicine and Epidemiology, Tohoku Medical Megabank Organization, 2-1, Seiryo-machi, Aoba-ku, 980-8573 Sendai, Miyagi Japan; 10 Division of Molecular Epidemiology, Tohoku University Graduate School of Medicine, 2-1 Seiryo-machi, Aoba-ku, 980-8573 Sendai, Miyagi Japan

**Keywords:** Clinical services, Drug information, Drug summary, Information systems and technology, Tracing reports

## Abstract

**Background:**

Information sharing among medical professionals is important for providing quality medical care. The purpose of the present study was to elucidate the actual status of information sharing between hospitals and other healthcare delivery facilities by surveying information sharing among the pharmaceutical departments of Japanese hospitals in 2020 conducted by the Japanese Society of Hospital Pharmacists.

**Methods:**

Responses were received from 3612 (43.6%) of the 8278 target medical institutions between May 2020 and August 2020.

**Results:**

The proportions of hospitals that shared information with community pharmacies, other hospitals, and long-term nursing homes were 40.6%, 36.4%, and 27.3%, respectively. While tracing reports were the most common tool used by hospitals for information sharing with community pharmacies (54.3%), drug summaries were used for sharing information with other hospitals and long-term nursing homes (77.4% and 78.0%, respectively). The proportion of hospitals sharing information with community pharmacies and other hospitals showed a tendency to increase as the number of hospital beds increased. No relationship could be established between the number of hospital beds and the proportion of hospitals sharing information with long-term nursing homes.

**Conclusion:**

Information between hospitals and community pharmacies was shared primarily using tracing reports, whereas information between hospitals and other hospitals and long-term nursing homes was primarily shared via drug summaries.

## Background

For providing high-quality medical care and a community-based integrated care system, information has to be shared timely and appropriately between relevant personnel, including medical/caregiving service providers, and information and communication technology (ICT) has to be used effectively [1]. Furthermore, a well-balanced medical and long-term care service delivery system suitable for the target area should be established [1]. In Japan, the population over 65 years will reach approximately 36.8 million in 2025 and will account for 30% of the total population [2]. However, the system for sharing information on this population in both medical and caregiving domains remains inadequate.

Tracing reports and drug summaries are typical tools used by hospital pharmacists to share information with other medical care facilities, and the usefulness of these sources for information sharing is well established [3–7]. Tokumaru et al. [3] evaluated the usefulness of information from tracing reports of community pharmacies involved in outpatient cancer chemotherapy and reported that 73.7% of the information feed back using tracing reports led to changes in pharmacotherapy. Tracing reports are often used for reporting from community pharmacies to hospitals [8, 9]. However, the usefulness of information sharing using tracing reports from hospital pharmacists to community pharmacies has also been reported [6]. Suzuki et al. [6] shared information with community pharmacies using inter-facility information communication forms for proper drug use. They reported that this initiative contributed to the efficiency of information collection and improved the quality of medication guidance at community pharmacies [6]. Takai et al. [7] investigated variations in the number of heart disease events in patients with cardiovascular disease before and after providing drug summaries to community pharmacies. This study identified that the number of heart disease events was significantly reduced in the patient group for which drug summaries had been provided to the community pharmacy (intervention group) when compared to those in the patient group before the provision of drug summaries was introduced (control group). In addition, a study comparing scenarios before and after the provision of drug summaries using the Morisky Medication Adherence Scale-4 (an index of medication adherence) identified no significant change in medication adherence in the intervention group [7]. However, six months after registration, a significant deterioration in medication adherence was noted in the control group. Thus, tracing reports and drug summaries contribute to the visualization of information related to high-quality medical care provision systems and long-term care indicated by the “Basic Policy for Comprehensively Securing Medical Care and Long-term Care in the Community,“ which has been formulated by the Ministry of Health, Labour and Welfare, Japan [1].

The usefulness of tracing reports and drug summaries [3–7] has been described only in reports focusing on large hospitals, and suitable information provision methods may vary depending on the characteristics of the hospital in question. However, the actual use of these methods in large hospitals and other small and medium-sized hospitals is unknown. Hence, the present study aimed to elucidate the actual situation of information sharing between medical care providers by using the response data of a survey conducted by the Japanese Society of Hospital Pharmacists (JSHP) on the status of information sharing among the pharmaceutical departments of Japanese hospitals in 2020. The results obtained here will help establish a nationwide information provision system common to all medical facilities in the future.

## Methods

### Survey design

The present study utilized a questionnaire-based survey on the status of information sharing among the pharmaceutical departments of Japanese hospitals in 2020 conducted by the JSHP. Participants were able to take the survey online or send their responses via email. In addition, unless otherwise specified, the survey requested a response regarding their departments’ information sharing situation as of June 1, 2020. The survey was conducted from May 25, 2020, to August 31, 2020. The facilities included in the survey were medical institutions in Japan, including those without a member affiliated with the JSHP.

### Sharing of patient information with other healthcare delivery facilities

**Fig. 1:**
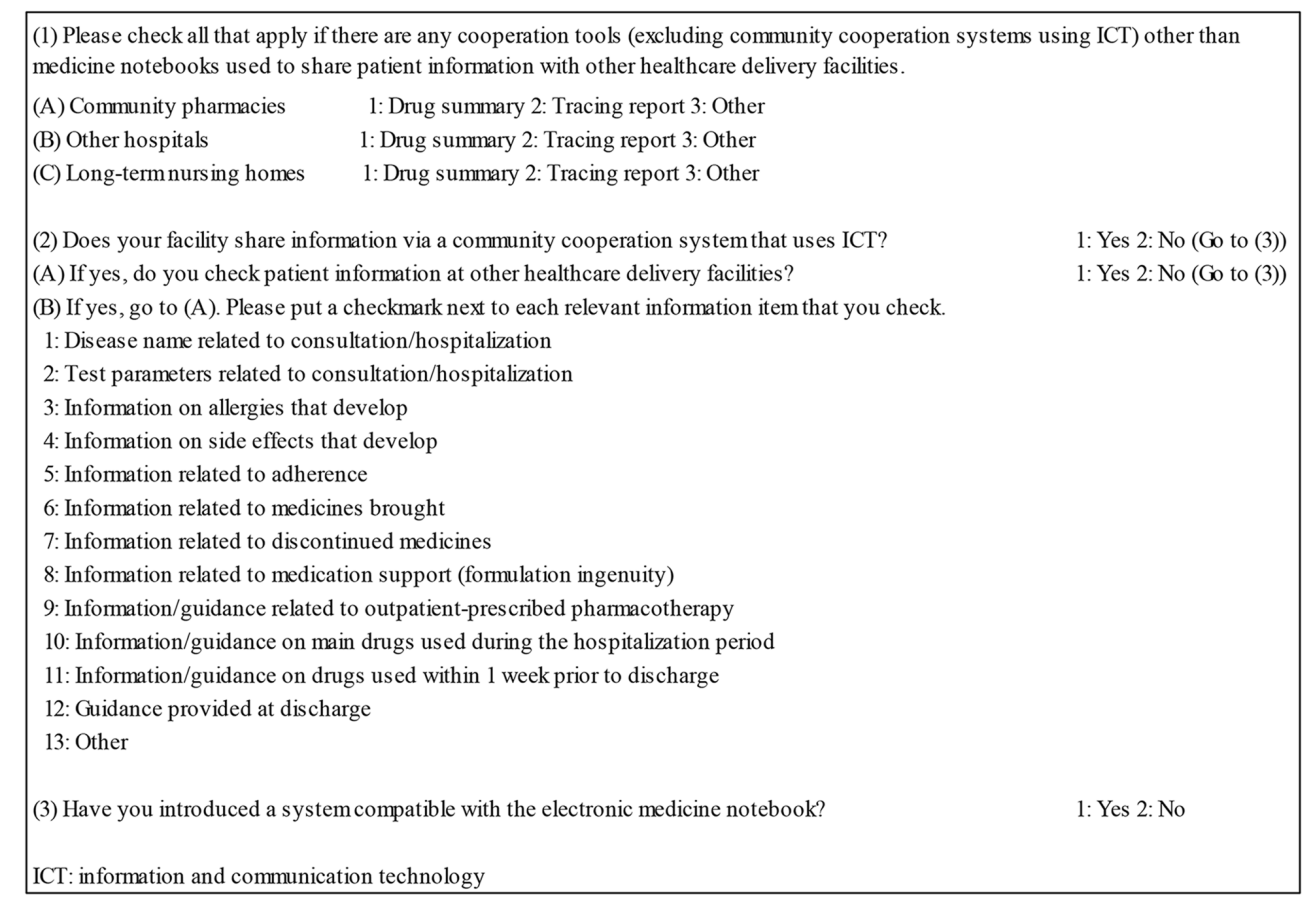
Information collected from the survey on the status of information sharing among the pharmaceutical departments of Japanese hospitals in 2020

The questions regarding sharing of information with other healthcare delivery facilities (Fig. 1) inquired about the tools for sharing patient information, the presence or absence of community collaboration systems using ICT, patient information shared within the system, and whether or not there were electronic medicine notebooks (booklets to record prescription history). Whenever relevant choices were selected in response to the checkbox-form questions on the use of drug summaries and tracing reports (excluding medicine notebooks and community collaboration systems using ICT) as ways to share information between the home facility and other healthcare delivery facilities (community pharmacies, other hospitals, and long-term nursing homes), it was considered as sharing of information between healthcare delivery facilities.

### Drug summary

A drug summary [10] is a document prepared not only for hospital transfers but also for long-term care and welfare facilities. Drug summaries describe medications that require attention with respect to test results, such as those pertaining to renal function, and are necessary to ensure adequate pharmaceutical care while in the hospital. These summaries include information for both medications brought upon admission and prescriptions at discharge. In general, they are provided by hospitals to other medical institutions.

### Tracing reports

A tracing report [8, 9] is a document for providing feedback that is considered “desirable to be provided to prescribing doctors,“ including medication adherence status and use of health foods as reported by patients at community pharmacies. However, information transfer speed is not prioritized by the use of tracing reports. In general, community pharmacists produce tracing reports, which are then provided to prescribing hospitals.

### Statistical analysis

Of the facilities that responded to the survey, only those with one or more full-time equivalent pharmacists, and 20 beds or more, were analyzed. According to the Medical Care Act [10], institutions with less than 20 beds are classified as clinics; hence, they are not considered hospitals. In addition, hospitals with less than one full-time equivalent pharmacist may be inadequate for the analysis of pharmacists’ efficiency and job performance. Therefore, facilities with less than 20 beds and less than one full-time equivalent pharmacist were excluded from the present study.

The number of full-time equivalent pharmacists is the number of full-time pharmacists plus the number of part-time pharmacists converted to full-time pharmacists. The number of part-time pharmacists converted to full-time pharmacists was calculated using the following formula:

The number of part-time pharmacists converted to full-time pharmacists =

$$\frac{\begin{gathered}{\text{Number of days per week worked by part} - \text{time pharmacists} ~ \times}\\ \text{working hours} \times \text{number of part} - \text{time pharmacists} \end{gathered}} {\text{Full} - \text{time pharmacist working hours per week}}$$

The number of hospital beds was categorized by referring to the survey in a 2018 report by the JSHP [11]: 20–49, 50–99, 100–299, 300–499, and ≥ 500 beds. The relationship between the number of beds and the frequency of information sharing, the usage of each tool for information sharing, information sharing with the community cooperation system using ICT, and the introduction of systems compatible with electronic medicine notebooks, were evaluated using the Cochran-Armitage propensity test. R version 4.1.2 (R Foundation for Statistical Computing, Vienna, Austria) was used for statistical analyses, and the significance level was set at 5%.

### Ethical considerations

The present study was conducted with the approval of the Tohoku Medical Megabank Organization Ethics Committee, Tohoku University (Approval No. 2021-4-074).

## Results

**Fig. 2:**
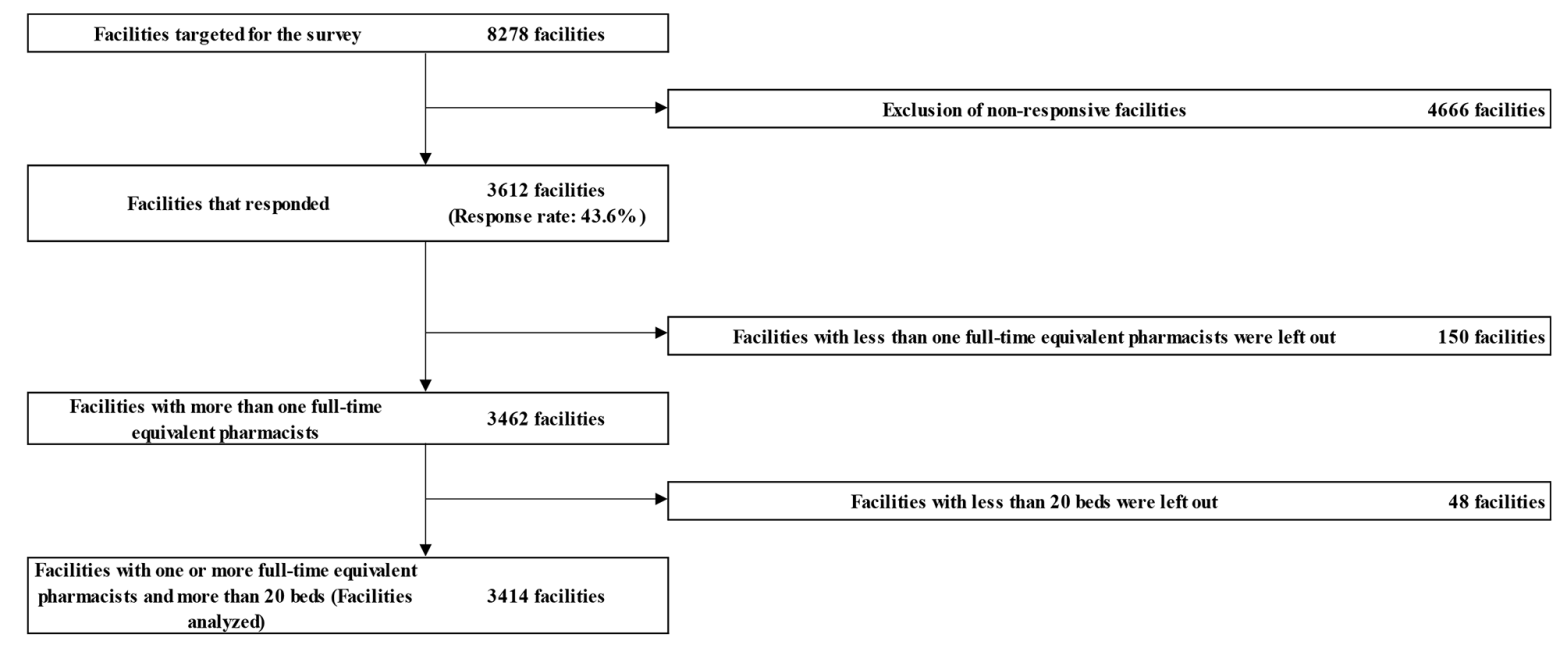
Flow diagram showing the facilities targeted for the survey and facilities included in the final analysis

The survey recruited 8278 facilities but only 3612 responded (response rate of 43.6%). Excluded from the analysis were 150 facilities that had less than one full-time equivalent pharmacist and 48 facilities with less than 20 beds. Thus, 3414 facilities were analyzed (41.2%) (Fig. 2). The number of beds and full-time equivalent pharmacists in these 3414 facilities is outlined in Table 1.

**Table 1:** Number of beds and full-time equivalent pharmacists in the analyzed facilities (n = 3414)

	20–49 beds	50–99 beds	100–299 beds	300–499 beds	≥ 500 beds	Total
**1 pharmacist, n (%)**	109 (55.6)	178 (30.3)	72 (4.6)	0 (0.0)	0 (0.0)	359
**2–4 pharmacists, n (%)**	78 (39.8)	363 (61.7)	793 (50.5)	102 (14.2)	6 (1.7)	1342
**5–9 pharmacists, n (%)**	9 (4.6)	44 (7.5)	472 (30.1)	133 (18.5)	21 (6.1)	679
**10–19 pharmacists, n (%)**	0 (0.0)	3 (0.5)	204 (13.0)	250 (34.9)	32 (9.3)	489
**≥ 20 pharmacists, n (%)**	0 (0.0)	0 (0.0)	28 (1.8)	232 (32.4)	285 (82.9)	545
**Total**	196	588	1569	717	344	3414

The responses related to information provision according to the number of beds are outlined in Table 2. The average out-of-hospital prescription issuance rate was the lowest (64.4%) in hospitals with 20–49 beds and the highest (79.7%) in hospitals with more than 500 beds (Table 2 − 1). In total, 1690 facilities (49.5%) had been sharing patient information with community pharmacies, other hospitals, or long-term nursing homes via drug summaries and tracing reports (excluding medicine notebooks and community collaboration systems using ICT) (Table 2A). Of the 1387 facilities (40.6%) that had been sharing information with community pharmacies, 753 (54.3%) and 701 (50.5%) facilities used tracing reports and drug summaries, respectively, to share information (Table 2B). There were 1241 facilities (36.4%) that had been sharing information with other hospitals; 960 (77.4%) used drug summaries to share information, which was the highest proportion noted (Table 2C). A total of 933 facilities (27.3%) shared information with long-term nursing homes, 728 of which (78.0%) used drug summaries to share information, which was the highest proportion noted (Table 2D). Overall, other information sharing tools besides drug summaries and tracing reports included telephone calls, facsimiles, drug information forms, medical referral letters, and nursing summaries. When focusing only on tracing reports at facilities where information was shared, hospitals with 500 or more beds had the highest proportion of tracing report use among hospitals with different numbers of beds. Similarly, when focusing only on drug summaries at facilities where information was shared, hospitals with 20–49 beds had the highest proportion of drug summary use. Concerning the relationship between information sharing and its tools according to the facility’s number of beds, there was a positive correlation between the number of beds and information sharing with community pharmacies and other hospitals (*p* < 0.0001 and *p* = 0.007, respectively). No trend was observed between the number of beds and information sharing with long-term nursing homes (*p* = 0.07).

**Table 2:** Out-of-hospital prescription rates and responses to information provision according to the number of hospital beds

		Number of beds, n						
		20–49 beds	50–99 beds	100–299 beds	300–499 beds	≥ 500 beds	Total	p-value^*^
		n = 196	n = 588	n = 1569	n = 717	n = 344	n = 3414	
(1) Average out-of-hospital prescription issuance rate (%)		64.4	65.0	67.6	76.1	79.7	70.4	-
(2) Cooperation tool (except community cooperation systems using ICT) for sharing patient information with other healthcare delivery facilities								
	(2-A) Information shared with community pharmacies, other hospitals, or long-term nursing homes, n (%)	64 (32.7)	247 (42.0)	719 (45.8)	439 (61.2)	221 (64.2)	1690 (49.5)	< 0.0001
	(2-B) Information shared with community pharmacies (corresponding to 2-B-1 to 2-B-3), n (%)	38 (19.4)	168 (28.6)	580 (37.0)	393 (54.8)	208 (60.5)	1387 (40.6)	< 0.0001
	If “Yes” to the above, the method of sharing information is:							
	(2-B-1) Drug summary, n (%)	24 (63.2)	103 (61.3)	333 (57.4)	168 (42.7)	73 (35.0)	701 (50.5)	< 0.0001
	(2-B-2) Tracing report, n (%)	10 (26.3)	52 (31.0)	273 (47.1)	260 (66.2)	158 (76.0)	753 (54.3)	< 0.0001
	(2-B-3) Other, n (%)	6 (15.8)	29 (17.3)	67 (11.6)	36 (9.2)	32 (15.4)	170 (12.3)	0.39
	(2-C) Information shared with other hospitals (corresponding to 2-C-1 to 2-C-3), n (%)	54 (27.6)	211 (35.9)	555 (35.4)	293 (40.9)	128 (37.2)	1241 (36.4)	0.007
	If “Yes” to the above, the method of sharing information is:							
	(2-C-1) Drug summary, n (%)	43 (79.6)	164 (77.7)	435 (78.4)	223 (76.1)	95 (74.2)	960 (77.4)	0.30
	(2-C-2) Tracing report, n (%)	4 (7.4)	18 (8.5)	74 (13.3)	46 (15.7)	26 (20.3)	168 (13.5)	0.0005
	(2-C-3) Other, n (%)	10 (18.5)	42 (19.9)	92 (16.6)	54 (18.4)	22 (17.2)	220 (17.7)	0.72
	(D) Information shared with long-term nursing homes (corresponding to 2-D-1 to 2-D-3), n (%)	36 (18.4)	162 (27.6)	427 (27.2)	219 (30.5)	89 (25.9)	933 (27.3)	0.07
	If “Yes” to the above, the method of sharing information is:							
	(2-D-1) Drug summary, n (%)	30 (83.3)	124 (76.5)	335 (78.5)	170 (77.6)	69 (77.5)	728 (78.0)	0.79
	(2-D-2) Tracing report, n (%)	2 (5.6)	13 (8.0)	52 (12.2)	25 (11.4)	11 (12.4)	103 (11.0)	0.19
	(2-D-3) Other, n (%)	5 (13.9)	34 (21.0)	66 (15.5)	35 (16.0)	14 (15.7)	154 (16.5)	0.47
(3) Does your facility share information via a community cooperation system using ICT?								
	Yes, n (%)	13 (6.6)	28 (4.8)	165 (10.5)	116 (16.2)	90 (26.2)	412 (12.1)	< 0.0001
(3-A) If “Yes” to the above, do you check patient information at other healthcare delivery facilities?								
	Yes, n (%)	7 (53.8)	17 (60.7)	76 (46.1)	32 (27.6)	26 (28.9)	158 (38.3)	< 0.0001
(3-B) If “Yes” to the above, place a checkmark next to each relevant information item.								
	(3-B-1) Disease name related to consultation/hospitalization, n (%)	6 (85.7)	17 (100.0)	59 (77.6)	24 (75.0)	18 (69.2)	124 (78.5)	0.04
	(3-B-2) Test parameters related to consultation/hospitalization, n (%)	3 (42.9)	9 (52.9)	58 (76.3)	18 (56.3)	16 (61.5)	104 (65.8)	0.96
	(3-B-3) Information on allergies that develop, n (%)	3 (42.9)	15 (88.2)	47 (61.8)	21 (65.6)	14 (53.8)	100 (63.3)	0.37
	(3-B-4) Information on side effects that develop, n (%)	2 (28.6)	13 (76.5)	39 (51.3)	15 (46.9)	14 (53.8)	83 (52.5)	0.18
	(3-B-5) Information related to adherence, n (%)	1 (14.3)	10 (58.8)	19 (25.0)	10 (31.3)	10 (38.5)	50 (31.6)	0.84
	(3-B-6) Information related to medicines brought, n (%)	6 (85.7)	17 (100.0)	59 (77.6)	20 (62.5)	16 (61.5)	119 (75.3)	0.003
	(3-B-7) Information related to discontinued medicines, n (%)	1 (14.3)	12 (70.6)	38 (50.0)	16 (50.0)	12 (46.2)	79 (50.0)	0.94
	(3-B-8) Information related to medication support (formulation ingenuity), n (%)	2 (28.6)	9 (52.9)	24 (31.6)	13 (40.6)	9 (34.6)	57 (36.1)	0.86
	(3-B-9) Information/guidance related to outpatient-prescribed pharmacotherapy, n (%)	3 (42.9)	7 (41.2)	30 (39.5)	13 (40.6)	10 (38.5)	63 (39.9)	0.86
	(3-B-10) Information/guidance on main drugs used during the hospitalization period, n (%)	5 (71.4)	11 (64.7)	45 (59.2)	18 (56.3)	14 (53.8)	93 (58.9)	0.32
	(3-B-11) Information/guidance on drugs used within 1 week prior to discharge, n (%)	3 (42.9)	9 (52.9)	30 (39.5)	8 (25.0)	9 (34.6)	59 (37.3)	0.15
	(3-B-12) Guidance provided at discharge, n (%)	0 (0.0)	3 (17.6)	19 (25.0)	6 (18.8)	5 (19.2)	33 (20.9)	0.71
	(3-B-13) Other, n (%)	0 (0.0)	0 (0.0)	2 (2.6)	1 (3.1)	4 (15.4)	7 (4.4)	0.01
(4) Have you introduced a system compatible with the electronic medicine notebook?								
	Yes, n (%)	3 (1.5)	15 (2.6)	51 (3.3)	29 (4.0)	27 (7.8)	125 (3.7)	< 0.0001

^*^The relationship between the number of beds and the proportion of information sharing, use of each tool to share information, information sharing using the community cooperation system via ICT, and introduction of systems compatible with the electronic medicine notebooks was evaluated using the Cochran-Armitage propensity test

ICT: information and communication technology

It was found that 412 facilities (12.1%) used ICT to share information via the community cooperation system (Table 2). Of these 412 facilities, 158 (38.3%) checked patient information of other healthcare delivery facilities through ICT. In 124, 119, and 104 of these 158 facilities (78.5%, 75.3%, and 65.8%, respectively), the most frequently checked patient information was disease name (related to consultation/hospitalization), medications brought by the patients, and test parameters related to consultation/hospitalization, respectively.

There were 125 facilities (3.7%) that had introduced a system compatible with electronic medicine notebooks, and this proportion was the highest in hospitals with 500 or more beds (Table 2).

## Discussion

The present study aimed to elucidate the actual situation of information sharing between medical care providers by using the data from a survey on the status of information sharing among the pharmaceutical departments of Japanese hospitals in 2020 conducted by the JSHP. We found that 49.5% of the facilities analyzed shared patient information with community pharmacies, other hospitals, or long-term nursing homes by using drug summaries and tracing reports. Tracing reports accounted for more than half (54.3%) of the tools for patient information sharing with community pharmacies. On the other hand, < 80% of patient information sharing with other hospitals and long-term nursing homes occurred through the use of drug summaries and approximately 12% via tracing reports. As mentioned above, the tools used to share information varied, depending upon whether the information was being shared with community pharmacies or other facilities. Results of previous studies [3–6] and the use of tracing reports by various facilities [8, 9] also indicate that information sharing via tracing reports often occurs between hospitals and community pharmacies. The Community Cooperation Pharmacy Certification System (based on the revision of the “Act on Securing Quality, Efficacy, and Safety of Products Including Pharmaceuticals and Medical Devices” in 2019 [12]) has been in force since August 2021. Under this system, community cooperation pharmacies are required to handle and continuously coordinate centralized medication information with other healthcare delivery facilities not only throughout outpatient visits but also during medical care provision at home and at the times of admission and discharge. As the number of community pharmacies increases, the proportion of tracing reports being used as a tool for patient feedback from community pharmacies to hospitals may also increase as a result. The frequent use of drug summaries compared with that of tracing reports for information sharing with other hospitals and long-term nursing homes could be attributed to the fact that summaries are prepared during admission and transfer to long-term nursing homes. Drug summaries are prepared not only at the time of patients’ admission to hospitals but also while considering possible transfers to long-term nursing homes and welfare facilities. Therefore, they include a column to enter information on medicines brought by patients and information necessary for practicing pharmaceutical care, in addition to prescriptions at discharge [13]. Such circumstances may explain the frequent use of drug summaries compared with that of tracing reports for patient information sharing with other hospitals and long-term nursing homes. In our study, when focusing only on tracing reports at facilities where information was shared, hospitals with 500 or more beds had the highest proportion of tracing report use among hospitals with different numbers of beds. Similarly, when focusing only on drug summaries at facilities where information was shared, hospitals with 20–49 beds had the highest proportion of drug summary use. In small-scale hospitals, which have a low frequency of issuing out-of-hospital prescription, information sharing regarding pharmaceuticals often occurs within the facility. Therefore, the proportion of tracing reports, which are mainly used as tools for sharing information with community pharmacies [8, 9], is considered to be low. Hence, it is conceivable that the proportion of drug summary use will be higher in medium to small-scale hospitals that have low out-of-hospital prescription issuance rates, whereas the proportion of tracing report use will be higher in large-scale hospitals that have high out-of-hospital prescription issuance rates. Additionally, in some cases, a pharmacist belonging to the drug information (DI) department needs to be available to manage tracing reports [8, 9]. However, due to the limited number of staff in medium-to-small-scale hospitals, pharmacists belonging to the DI department may not be able to perform DI duties adequately due to a workload burden. In the present study, we did not observe any trends with regard to the sharing of information between hospitals and long-term nursing homes, regardless of hospital size. It is, therefore, necessary that future studies investigate information sharing between hospitals and long-term nursing homes in more detail.

Facilities that were sharing information using the community cooperation system via ICT accounted for 12.1% of all facilities. Additionally, the use of ICT increased as the number of hospital beds increased. According to a report by the Ministry of Health, Labour and Welfare, large-scale hospitals have a higher proportion of ICT use in the form of tools such as electronic medical records and ordering systems [14]. Large-scale hospitals deal with more patient information than small-scale hospitals, potentially attributed to their increased proportion of ICT use to improve the efficiency of information processing. Meanwhile, compared to large-scale hospitals, a higher proportion of small-scale hospitals had access to patient information from other healthcare providers. Patients may be referred to large-scale hospitals, such as university hospitals, for complex surgeries or advanced medical treatments that are difficult to perform at small- and medium-scale hospitals. After treatment or surgery, patients may return to small-scale hospitals for recovery. In fact, the referral rate from other hospitals is higher at university hospitals than the reverse referral rate [15]. In the present study, the assessment of the status of surgery and treatment which occurred at large-scale hospitals may have contributed to the higher rate of access to patient information. The results of the present study also revealed that, compared to large-scale hospitals, small-scale hospitals more frequently access patient information including the patients’ disease and treatment details (prescription intention). Small-scale hospitals that did not perform actual surgery or treatment lacked information regarding the disease type and treatment status, and thus they may access this information more frequently than large-scale hospitals. In the present study, we did not investigate the medical facilities accessed by each hospital, warranting future studies to investigate this issue. The most frequently assessed patient information was the specific disease of each patient and the test parameters related to the consultation/hospitalization, as well as information related to medicines consumed by the patients. Patient information is considered relatively easy to obtain when a patient is scheduled to be admitted to the home facility or being admitted by referral. On the other hand, healthcare providers find it difficult to obtain important patient information such as the patients’ disease, test parameters, and medications in patients admitted urgently, or severely ill patients who are unable to communicate. Hence, information on disease names, test parameters, and information on medications brought by patients were those most frequently collected using ICT. In addition, the Japanese government is promoting the utilization of ICT in the medical field [1]. However, only 3.7% of the facilities described in the present study have introduced systems compatible with electronic medicine notebooks, which is similar to the low proportion of facilities using the community cooperation system with ICT for information sharing. A previous survey-based study of 302 community pharmacies revealed that only 2% were using ICT networks as a tool for sharing information with hospitals or clinics [16]. Although there have been previous reports on the usefulness of ICT in the medical domain, such as the backup system for patient clinical information using the Miyagi Medical and Welfare Information Network after the Great East Japan Earthquake [17] and the Kibitan Health Net to ascertain HbA1c in diabetes patients [18], the initial start-up costs and necessary maintenance costs associated with introducing ICT systems may explain why it is not as widely used.

The present study had several limitations. Firstly, there is the possibility of selection bias as the present study analyzed facilities that participated in the survey; their decision to participate may have been due to the pharmacy’s current situation being of interest. Secondly, medicine notebooks were excluded as a tool for sharing patient information in the fact-finding survey. Although the medicine notebook is an important tool for information sharing, the data used in the present study was secondarily used from the survey already conducted by the JSHP; hence, it was not possible to add the medicine notebook as an information sharing tool. Kimoto et al. [19], in the Aizu Pharmaceutical Cooperation Council, reported that it was possible to share drug and medical information in the emergency medical care community by using medicine notebooks common to the community (the “Aizu Drug Notebook”). In addition, a questionnaire survey on inter-pharmacy cooperation and medicine notebooks was conducted for 112 community pharmacists (response rate: 100%), and the highest proportion of respondents, 71.4%, reported that medicine notebooks are appropriate for inter-pharmacy cooperation. Therefore, as the survey excluded medicine notebooks as a tool to share patient information, our study may have underestimated the proportion of information sharing between hospitals and community pharmacies. Thirdly, as the survey only had questions about whether or not drug summaries and tracing reports were used as tools to share information, it is not clear what kind of patient information was being shared using these tools. Fourthly, as the survey was limited to the pharmaceutical departments of medical and care facilities, our study may not account for cooperation between hospitals and long-term nursing homes that are not mediated by pharmacists. Finally, the survey could not account for the direction of information sharing, whether it was from the home facility to other facilities or vice versa. Nevertheless, the findings of the present study are useful in examining the future of information sharing and ICT utilization in the community health care system. The present study revealed differences in the information sharing tools, which were dependent on the number of hospital beds, the medical institution sharing the information, and the rate of issuance of out-of-hospital prescriptions. Therefore, it is necessary to standardize information sharing tools and content of information for each distinct hospital size, and to develop tools that are easy to operate for each, including the incorporation of medicine notebooks.

## Conclusion

Our study elucidated how patient information is shared between medical and care facilities. In particular, information sharing between hospitals and community pharmacies was primarily carried out using tracing reports, whereas that between hospitals and other hospitals and long-term nursing homes occurred via drug summaries. In addition, the scale of the facility (number of beds) was related to the sharing of information with community pharmacies and other hospitals. On the other hand, we established no link between the scale of the facility and the sharing of information with long-term nursing homes. In future studies, we aim to investigate the type of information sought by facilities during information sharing. Additionally, we anticipate that an improved cooperative setup can be developed for medical care and long-term care facilities by improving the convenience of using various tools to share information. In addition, we believe that the widespread use of ICT-based information sharing systems in many facilities will improve the efficiency of information sharing in future.

## Data Availability

Not applicable.

## Abbreviations

DI: Drug information.
ICT: Information and communication technology.
JSHP: Japanese Society of Hospital Pharmacists.
